# The isolation of rumen enterococci strains along with high potential utilizing cyanide

**DOI:** 10.1038/s41598-023-40488-9

**Published:** 2023-08-14

**Authors:** Waroon Khota, Chatchai Kaewpila, Thachawech Kimprasit, Wasan Seemakram, Suntorn Kakaisorn, Metha Wanapat, Anusorn Cherdthong

**Affiliations:** 1 Department of Animal Science, Faculty of Agriculture, Khon Kaen University, Khon Kaen, 40002 Thailand; 2 Department of Animal Science, Faculty of Natural Resources, Rajamangala University of Technology Isan, Sakon Nakhon, 47160 Thailand; 3 Department of Microbiology, Faculty of Science, Khon Kaen University, Khon Kaen, 40002 Thailand; 4 Animal Feed Inter Trade Co. Ltd., Khon Kaen, 40002 Thailand

**Keywords:** Microbiology, Zoology

## Abstract

Cyanogenic glycosides in forage species and the possibility of cyanide (CN) poisoning can have undesirable effects on ruminants. The literature estimates that unknown rumen bacteria with rhodanese activity are key factors in the animal detoxification of cyanogenic glycosides, as they are capable of transforming CN into the less toxic thiocyanate. Therefore, identifying these bacteria will enhance our understanding of how to improve animal health with this natural CN detoxification process. In this study, a rhodanese activity screening assay revealed 6 of 44 candidate rumen bacterial strains isolated from domestic buffalo, dairy cattle, and beef cattle, each with a different colony morphology. These strains were identified as belonging to the species *Enterococcus faecium* and *E. gallinarum* by 16S ribosomal DNA sequence analysis. A CN-thiocyanate transformation assay showed that the thiocyanate formation capacity of the strains after a 12 h incubation ranged from 4.42 to 25.49 mg hydrogen CN equivalent/L. In addition, thiocyanate degradation resulted in the production of ammonia nitrogen and acetic acid in different strains. This study showed that certain strains of enterococci substantially contribute to CN metabolism in ruminants. Our results may serve as a starting point for research aimed at improving ruminant production systems in relation to CN metabolism.

## Introduction

Reducing toxic substances is important to support the health and productivity of ruminant livestock^1–3^. Cyanide (CN), found in the form of cyanogenic glycosides in tropical regions, is highly toxic when ingested due to its ability to prevent normal oxygen metabolism, resulting in severe consequences such as histotoxic anoxia and death^4^. CN compounds are present in primary forage species such as cassava and various sorghum varieties^5^. Generally, these toxic plants require a processing phase to render them suitable for livestock consumption^6,7^. The lethal dose of hydrogen CN (HCN) for ruminant livestock such as cattle and sheep is approximately 2 mg/kg body weight^3^. Ruminants are more susceptible to CN poisoning than other livestock species such as swine and poultry^3^. The concentration of cyanogenic glycosides varies with plant breeds, planting management (including exposure to frost, drought, and herbicide application), growing stage, and animal feeding methods (e.g., grazing, cut and carry haymaking, and ensiling)^3^. If the concentration in the diet exceeds 400 mg/kg, it could pose a risk to ruminant animals^8^. Antidotes, including sodium thiosulphate and sodium nitrite, are recommended as the best option to reduce livestock losses when ruminants show signs of acute CN poisoning^3^.

The production of CN from cyanogenic glycosides in ruminant species is complex, as it depends on cyanogenesis and substantial detoxification by microbes and host ruminants. Cyanogenesis is the process whereby cyanogenic glycosides are hydrolyzed to HCN through several hydrolysis pathways involving ruminal alkalosis, plant enzymes, and microbial enzymes^9,10^. McSweeney et al.^1^ estimated that the after the formation of CN, the microbial transformation of CN to the less toxic thiocyanate could be the major CN detoxification process in the rumen; whereas CN degradation, which uses the carbon–nitrogen triple bond as its substrate and converts it into corresponding products such as formic acid, ammonia, and amino acids (asparagine or aspartate), may be a minor process.

Majak and Cheng^9^ demonstrated that rumen microbes primarily transform CN-rich substrates, such as cyanogenic glycosides and chemical CNs, to produce thiocyanate. Compared with CN degradation, thiocyanate formation is a substitution pathway catalyzed by the sulfurtransferase family of enzymes, mainly rhodanese (EC 2.8.1.1, thiosulfate: CN sulfurtransferase), which many eukaryotes and prokaryotes can produce^11^. Sharma et al.^12^ revealed that microbial rhodanese enzymes are mainly found in bacteria that can assimilate thiocyanate as an alternative nitrogen source. In ruminants, the levels of rhodanese activity are high in the rumen epithelium, hepatocytes, and kidney cortex, resulting in the production of thiocyanate before its excretion in urine^13^. Previous research has shown that adding rhodanese in the presence of CN improves rumen fermentation characteristics in vitro (e.g., increasing cumulative gas production, in vitro digestibility, and volatile fatty acid concentration)^14^ and in vivo (e.g., increasing propionate concentration)^15^.

To the best of our knowledge, the microbes responsible for CN detoxification in the rumen ecosystem remain unknown^16,17^. Identifying rumen microbial strains that contribute to thiocyanate formation will facilitate the development of methods to reduce the availability of free CN in the rumen and absorption through the gut wall, which would significantly alter the adaptation of ruminants to cyanogenesis. Therefore, the objectives of this study were to determine whether any strains of rumen bacteria are capable of detoxifying CN, and to assess their CN-thiocyanate transformation and degradation abilities using potassium CN (KCN) as a CN-rich substrate.

## Results

### Isolation, selection, and identification of rumen CN-detoxifying bacteria

**Figure 1:**
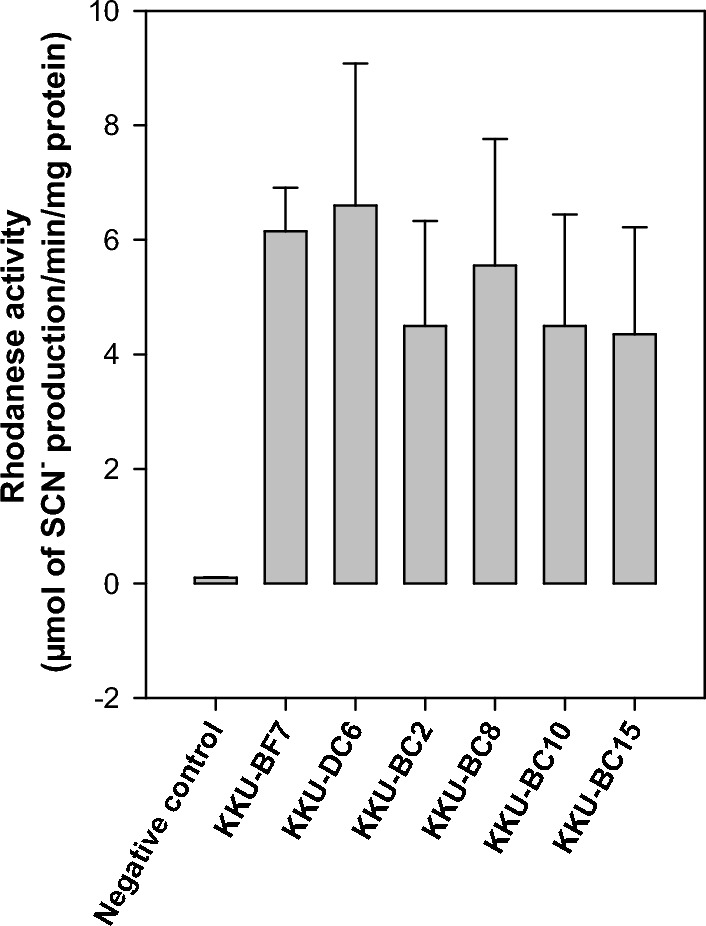
Rhodanese activity screening in rumen enterococci strains. SCN^−^, thiocyanate anion; error bar, standard deviation. In this rhodanese assay, the crude enzyme samples (n = 3) from each strain were extracted and incubated with the reaction mixture containing KCN. The negative control group (n = 3) consisted of rhodanese samples that were boiled to inactivate their activity. The rhodanese activity of the isolated strains ranged from 4.35 to 6.60 µmol of SCN^–^ production/min/mg protein.

**Figure 2:**
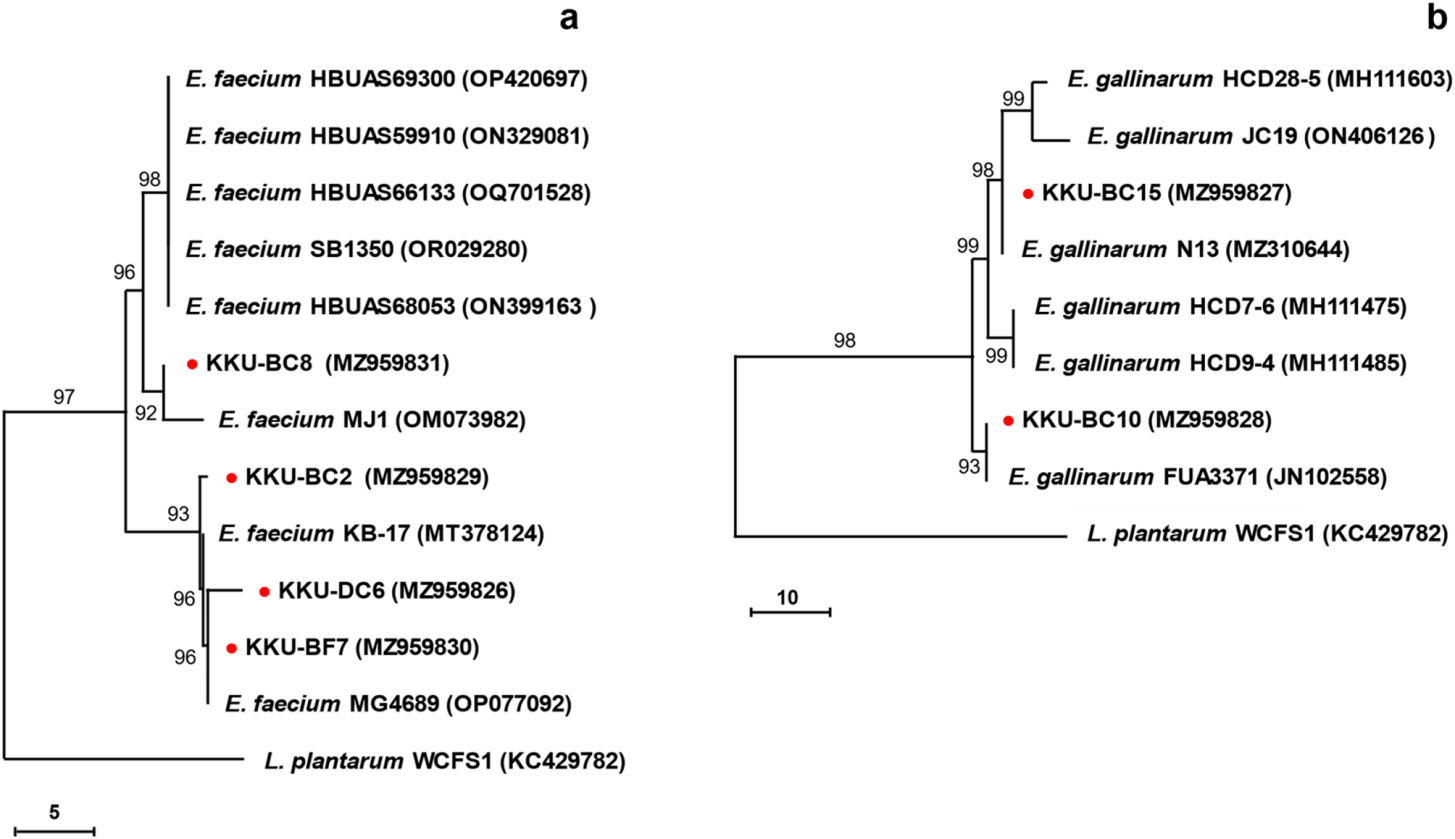
Phylogenetic trees of *E. faecium* (**a**) and *E. gallinarum* (**b**) type strains based on 16S rRNA gene sequences. The strains studied in our experiment are marked (red circle) and referred to the NCBI accession number. The nucleotide sequences were used to construct the highest log likelihood trees (− 14,901.92 for *E. faecium* and − 11,697.18 for *E. gallinarum*) with *Lactobacillus plantarum* WCFS1 (KC429782) as an outgroup. The trees were constructed using MEGA X, employing the Maximum Likelihood method and Tamura-Nei model, with 10,000 bootstrap iterations. The percentage of trees in which the associated taxa clustered together is shown next to the branches. Bars indicated sequence divergences.

Using a closed fermenter system, rumen bacteria that can survive in CN-rich substrate media were successfully isolated in 2020. We counted strains with different colony morphologies under a stereomicroscope at 40 × magnification. The selection was based on ruminant species and broad rhodanese activity. Six strains were archived with the following strain codes: KKU-BF7 from buffalo; KKU-DC6 from a dairy cow; and KKU-BC2, KKU-BC8, KKU-BC10, and KKU-BC15 from beef cattle. Rhodanese activity ranged from 4.35 to 6.60 µmol thiocyanate production/min/mg protein (Fig. 1). Based on 16S ribosomal DNA (rDNA) sequence analysis, the KKU-BF7, KKU-DC6, KKU-BC2, and KKU-BC8 strains were identified as *Enterococcus faecium* strain DSM 20,477 with percentage identity of 99.85%, 99.50%, 99.78%, and 99.85%, respectively. Furthermore, the KKU-BC10 and KKU-BC15 strains were closely related to *E. gallinarum* strain LMG 13,129 with percentage identify of 99.79% and 99.85%, respectively. Based on phylogenetic tree analyses using 16S rDNA sequences, the *E. faecium* strains in our study are closely identified with the related *E. faecium* strains obtained from the NCBI database and separated into two clades (Fig. 2a). The strains KKU-BC2, KKU-DC6, and KKU-BF7 are grouped together in the same clade, while the isolate KKU-BC8 is located in a separate clade. The two *E. gallinarum* strains in our study are divided into different clades (Fig. 2b). The strain KKU-BC10 is closely related to strain FUA3371, while the strain KKU-BC15 is highly similar to strain N13. The bootstrap values ranged from 92 to 99%.

### Characteristics of rumen CN-detoxifying bacteria

**Figure 3:**
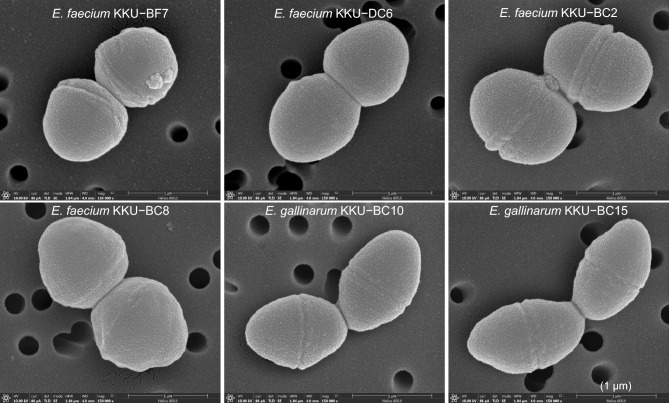
Scanning electron microscopy (SEM) at 150,000× magnifications of rumen enterococci strains. The image displays the cell morphology of pure strains. These bacteria have an ovoid shape with a cell diameter of approximately 1.0 µm.

Scanning electron microscopy showed a cell diameter of 1.0-µm for the six enterococci strains (Fig. 3). Biochemical testing revealed that the six enterococci strains were Gram-positive, catalase-negative, starch hydrolysis-negative, and incapable of producing hydrogen sulfide gas (Table 1). They were all facultative anaerobic and fermented sugars (ribose, glucose, lactose, and fructose), as well as amylose. In Lactobacilli de Man, Rogosa, Sharpe (MRS) broth, they all produced lactic acid (from 0.11 to 1.98 mmol/L) and acetic acid (from 5.34 to 7.61 mmol/L). The KKU-BF7 strain even produced a small amount of propionic acid. No strain produced butyric acid or valeric acid in this medium. Ammonia nitrogen production ranged from 0.38 to 3.91 mg/L.

**Table 1:** Characterization of rumen enterococci strains. OD_600_, optical density at 600 nm; − , negative test; + , positive test; np, not produced. Three analytical repetitions of each measurement were done for each strain.

Item	Enterococci strains					
	KKU-BF7	KKU-DC6	KKU-BC2	KKU-BC8	KKU-BC10	KKU-BC15
Gram staining	Positive	Positive	Positive	Positive	Positive	Positive
Catalase test	−	−	−	−	−	−
Starch hydrolysis test	−	−	−	−	−	−
Hydrogen sulfide gas production	−	−	−	−	−	−
Growth in MRS agar (24 h)						
Aerobic conditions	+	+	+	+	+	+
Anaerobic conditions	+	+	+	+	+	+
Carbohydrate fermentation (24 h)						
Ribose	+	+	+	+	+	+
Glucose	+	+	+	+	+	+
Lactose	+	+	+	+	+	+
Fructose	+	+	+	+	+	+
Amylose	+	+	+	+	+	+
Fermentation in MRS broth (24 h)						
Final pH	4.69	4.73	4.76	4.79	4.89	4.92
Cell density (OD_600_)	0.98	1.46	1.45	1.16	1.03	1.10
Lactic acid (mmol/L)	1.98	1.09	0.11	0.27	0.30	0.13
Acetic acid (mmol//L)	4.86	4.65	5.98	5.34	5.56	7.61
Propionic acid (mmol//L)	0.76	np	np	np	np	np
Butyric acid (mmol//L)	np	np	np	np	np	np
Valeric acid (mmol//L)	np	np	np	np	np	np
Ammonia nitrogen (mg/L)	0.38	3.59	3.91	3.51	3.03	3.81

**Figure 4:**
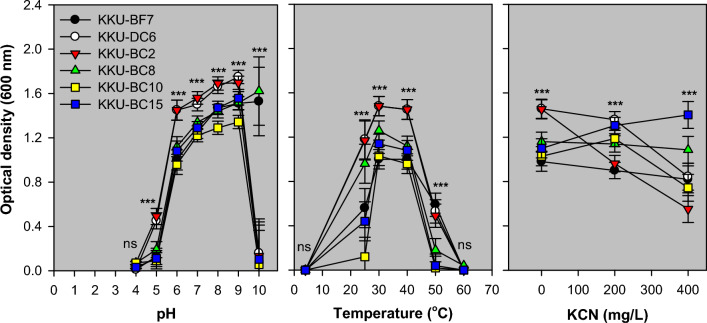
Growth at different pH, temperatures, and CN substrate concentrations in MRS broth after incubation for 24 h. Bar, standard error of the mean; ***, *p* < 0.001; ns, *p* > 0.05. The statistical difference among the six strains at each pH, temperature, or CN substrate concentration was analyzed using one-way ANOVA with three replicates. No strain grew at a pH of 4.0, and the optimal pH range for most strains was 6‒9. At a pH of 10.0, abundant growth of KKU-BF7 and KKU-BC8 strains was observed compared with the other four strains (*p* < 0.001). The bacteria did not grow at 4 °C and 60 °C. The optimal temperature range for most strains was 30‒40 °C, and KKU-DC6 and KKU-BC2 strains had an OD > 1.5, which was greater than any other strain (*p* < 0.001). The results showed that the susceptibility of the KKU-BC15 strain to CN was significantly lower than that of all other strains at high levels of CN (400 mg/L) (*p* < 0.001).

Figure 4 shows growth at different pH, temperatures, and CN substrate concentrations in MRS broth after incubation for 24 h. The strains grew well at pH 6.0–9.0 and temperature of 30–40 °C. The KKU-BF7 and KKU-BC8 strains were more resistant to alkaline conditions than the other six strains (*p* < 0.001). The KKU-BC15 strain grew well at a high KCN concentration (400 mg/L; *p* < 0.001).

### Capacity of strains to detoxify CN

**Figure 5:**
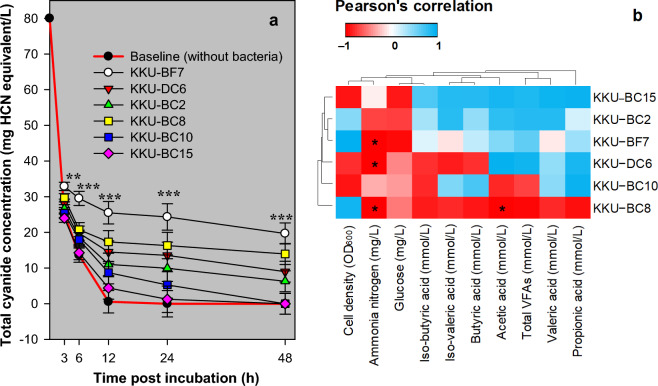
CN-thiocyanate transformation assay of rumen enterococci strains using an in vitro rumen fermentation technique. (**a**) Total CN concentration during the 48 h fermentation. Error bar, standard error of the mean; **, *p* < 0.01; ***, *p* < 0.001. (**b**) Pearson’s correlation heatmap of thiocyanate concentration and fermentation end-products for 12–48 h of incubation. The color scheme indicates the strength of correlation of thiocyanate degradation and fermentation end-products in each strain (n = 9). *, *p* < 0.05; OD_600_, optical density at 600 nm; VFAs, volatile fatty acids. In this assay, a cell-free rumen fluid medium containing KCN substrate was utilized to evaluate the ability of each strain to produce thiocyanate from the CN source. The fermentation process involved analyzing the CN compounds (KCN and thiocyanate) in the medium using a distillation method, and the results are reported in mg of HCN equivalent/L. The assimilation of thiocyanate within the cytoplasm of bacterial cells is thought to occur through their rhodanese activity. To differentiate thiocyanate from the remaining KCN substrate, a baseline reference (negative control without bacteria or a rhodanese source) was used during the incubation period. Compared with bacterial fermentation, the baseline KCN substrate releases free CN (HCN and CN^−^) instead of thiocyanate, as it possesses hydrolytic abilities in the medium. In (**a**), the total CN concentration in the medium was measured at 3, 6, 12, 24, and 48 h of incubation for each strain (n = 3) and baseline (n = 3). After 12 h of incubation, the baseline (plotted in the red line) showed a remarkably low concentration of CN from an initial concentration of 80 mg HCN equivalent/L. This suggests that there was minimal interference from the remaining KCN substrate on thiocyanate production after the strains’ rhodanese activity. The statistical difference among the six strains at each time of incubation was analyzed using one-way ANOVA with three replicates. The results indicated that the KKU-BF7 strain exhibited the highest capacity for CN-thiocyanate transformation, with a minimum of 25.49 mg HCN equivalent/L during the 12 h incubation period. This value was significantly greater than that of all other strains (*p* < 0.001). In (**b**) the fermentation end-products from each strain at 12, 24, and 48 h of incubation were observed to analyze their correlation with the thiocyanate values using Pearson’s correlation coefficient. The results indicate a significant association between the degradation of thiocyanate and the production of ammonia nitrogen (*p* < 0.05) and/or acetic acid (*p* < 0.05) in certain strains.

Figure 5a shows the CN-thiocyanate transformation capacities of the strains. This assay was performed without (as a baseline) and with bacteria in 80 mg HCN equivalent/L; the baseline condition showed the amount of CN substrate that remained in the medium. At 0 h of incubation (the starting point), the CN values between baseline and bacteria were similar. At the 12 h endpoint of the CN-thiocyanate transformation assay, the estimated amounts of thiocyanate formed by bacteria ranged from 4.42 to 25.49 mg HCN equivalent/L, with the KKU-BF7 strain outperforming the others (*p* < 0.001).

Figure 5b shows the linear correlations between thiocyanate degradation and fermentation (i.e., bacterial cell density, ammonia nitrogen, and volatile fatty acids, including the glucose substrate that remained). Degradation of thiocyanate by the KKU-BF7, KKU-DC6, and KKU-BC8 strains resulted in the production of ammonia nitrogen (*p* < 0.05). In the KKU-BC8 strain, thiocyanate degradation correlated with acetic acid production (*p* < 0.05). No correlation was found in the KKU-BC2, KKU-BC10, and KKU-BC15 strains (*p* > 0.05).

## Discussion

The genus *Enterococcus* is ubiquitous in various habitats and currently comprises 38 species^18^. In this study, phylogenic trees of 16S rRNA gene sequences indicated that the rumen isolates are closely related to the species *E. faecium* and *E. gallinarum* with bootstrap values ranging from 92 to 99% (Fig. 2a,b). It has previously been shown that the abundance of enterococci in the rumen is low, with ≤ 10^5^ colony forming units/mL^19^. Examples of species that can be isolated from rumen are *E. casseliflavus*, *E. gallinarum, E. faecium, E. cecorum*, *E. mundtii*, and *E. faecalis*^19–22^. Regarding the rumen ecosystem and fermentation process, an in vitro study demonstrated that the addition of *E. faecium* can effectively support the function of lactate-utilizing bacteria, thereby promoting the proliferation of rumen microbes and enhancing the synthesis of propionic acid^23^. Some strains of enterococci species can produce beneficial enzymes^24^, bacteriocin and enterocin^25^, and vitamin B12^26^. The rumen enterococci species might evolve themselves or receive a gene transferred from other organisms^11^. According to the estimated CN detoxification in rumen ecology, it should be a domain of CN‒thiocyanate transformation^1,9^.

This is the first study to report the CN-utilizing capacity of enterococci species in the rumen. The main CN-metabolizing properties of the isolated strains were determined based on the rhodanese activity of both crude enzyme extracts (Fig. 1) and whole cells (Fig. 5a). The enzyme facilitates the transfer of a sulfur atom from a donor molecule to an acceptor molecule^12^. In the Kyoto Encyclopedia of Genes and Genomes database (https://www.genome.jp/entry/R01931), sulfur metabolism uses thiosulfate and the CN ion to produce sulfite and thiocyanate. The capacity of microorganisms to metabolize CN using rhodanese has been observed in several strains of fungi and bacteria from diverse environments including *Fusarium* sp., *Azotobacter vinelandii*, *Bacillus brevis*, *Escherichia coli*, *Pseudomonas aeruginosa*, and *Thiobacillus* sp.^12^. However, rumen bacteria represent more than 31 genus groups^21^, with approximately 10^10^–10^11^ cells/mL and over 200 species, most of which are uncultured using standard techniques^27^. Approximately 80% of ruminal microbiota cannot be cultivated^27^, which naturally requires specific growth factors from the ecosystem of the rumen. Therefore, rumen bacterial species that can metabolize CN are not limited to only certain strains of enterococci. In a non-rumen environment, Gardner and Rawlings^28^ found that the levels of rhodanese activity obtained from a combination of bacteria cells present in bio-oxidation plants were 2–2.5 times higher than the activity observed in pure bacteria cultures. The range of rhodanese activity in the current study is in accordance with previous findings^28^. In addition, our results showed that the KKU-BF7 strain can produce a small amount of propionic acid (Table 1). Kim et al.^29^ suggested that some strains of *E. faecium* produce fumarate reductase, which reduces methanogenesis by producing propionic acid with fumarate conversion to succinate.

The results indicated that the bacteria grew within a pH range of 6–9 and a temperature range of 30–40 °C (Fig. 4). This finding suggests that the bacteria can be cultivated under a range of optimal conditions. However, the results showed that only some strains were resistant to a high CN concentration. These findings are similar to those of Prachumchai et al.^16^, which revealed that the numbers of rumen CN-utilizing bacteria decreased by 450 mg KCN/L. The results suggested that the KKU-BF7 strain was generally more active in CN-thiocyanate transformation than the five other strains (Fig. 5a). The ability of enterococci to degrade CN substrates was recently reported by López-Ramírez et al.^30^ based on the *E. hirae* KU175874 strain, which was isolated from gold processing plants. CN detoxification by microbes involves either degradation, transformation, or both biological processes with enzymes including cyanidase, CN dioxygenase, CN monooxygenase, nitrogenase, rhodanese, mercaptopyruvate sulfurtransferase, cyanide hydratase, CN dihydratase, nitrilase, nitrile hydratase, thiocyanate hydrolase, cyanoalanine synthase, and γ-cyano-α-aminobutyric acid synthase^12^. Thiocyanate degradation occurs in many microbial species^31^, which might use the β-carbonic anhydrase family of enzymes^32^, producing sulfur, carbon, and nitrogen substances^12^. According to the relative stability of CN compounds, the CN‒thiocyanate transformation assay used in this study could cause distinct losses due to the volatilization of free CN species and biological reactions from the simultaneous presence of the thiocyanate form of CN. Therefore, thiocyanate-degrading pathways can also be tested as additional processes in these bacteria. The results of this study showed that the degradation resulted in ammonia nitrogen and acetic acid production (Fig. 5b). The KKU-BC2 strain produced acetic acid in association with thiocyanate degradation, likely through an acetogenesis pathway.

Many enterococci-specific strains, isolated from various environments, have undergone extensive testing to assess their advantageous impacts on the livestock production system, including animals indirectly or directly fed bacteria^23,33^. Based on the findings of this study, future studies should test the therapeutic effects of the KKU-BF7 strain on ruminants receiving CN-containing plants. Compared with an antidote, successful probiotics depend on the synchronization of cyanogenesis and thiocyanate formation, which are fairly complex with several biological factors, especially the type and concentration of cyanogenic glycosides, cyanogenesis rate, mechanism of probiotics, and probiotic growth factors. Majak and Cheng^9^ demonstrated that CN levels can peak in the rumen approximately 30 min after consuming plants containing cyanogenic glycosides. The symptoms of CN poisoning in cattle can occur within hours or days^4^. Therefore, there is a possibility that high cyanogenesis under higher ruminal pH conditions or naturally high activity of microbial glucosidase will prevail over all other detoxifying mechanisms^9,10^. To restore optimal health conditions, rumen bacteria and host tissue possess distinct abilities to detoxify CN. A significant population of bacteria can be estimated to facilitate efficient detoxification processes within the rumen^16^. On the other hand, host tissue utilizes different pathways to detoxify and eliminate CN from the body^13^. Aminlari and Gilanpour^13^ reported that the epithelium of rumen, omasum, and reticulum are the richest sources of rhodanese in sheep and cattle. Thus, while rumen bacteria contribute significantly to CN utilization, host tissues also play a role in overall detoxification processes.

In conclusion, this is the first study to demonstrate that specific strains of enterococci play a role in the process of detoxifying CN in the rumen by increasing the synthesis of thiocyanate. Different strains of enterococci degrade thiocyanate differently, with some producing ammonia nitrogen and others producing acetic acid. These results will help fill a significant research gap in the field of nutritional ecology of ruminants. The use of bacteria as a probiotic therapeutic for ruminants receiving CN-containing plants requires more studies.

### Methods

All experiments were approved by the Animal Care and Use Committee of Khon Kaen University (Khon Kaen, Thailand), based on the Ethics of Animal Experimentation of the National Research Council, Thailand (Record No. IACUC-KKU-45/64). All analytical methods were carried out in accordance with relevant guidelines and regulations. The study was carried out in compliance with the ARRIVE guidelines. All reagents were of high purity grade. All KCN solutions were prepared with sterile deionized water and used immediately.

### Animal care and rumen fluid collection

The rumen fluid strains were obtained from mature non-pregnant female swamp buffalo (*Bubalus bubalis*), beef cattle (Thai-native × Brahman, *Bos indicus*), and Holstein dairy cows (*Bos taurus*) at Khon Kaen University in November 2020. Animals grazed on grasslands mainly containing Guinea grass, Ruzi grass, Signal grass, Verano, Leucaena, and Centro. In these grasslands, the donor animals were inadvertently subjected to small amounts of CN due to the presence of particular forage species and weeds known to generate CN such as Signal grass, Ruzi grass, and Giant sensitive plant. According to the findings of Euswas et al.^34^, the grasses contain less than 200 ppm CN, which is within safe limits for grazing. Napier grass, rice straw, and mineral bricks were supplemented in the barns. In addition, a concentrate consisting of cassava chips, rice bran, oil palm meal, soybean meal, cornmeal, urea, sulfur, salt, and premixed vitamins was provided daily at about 0.5–1.0% body weight. The animals were given free access to drinking water. Two heads of fresh rumen fluid (200 mL/head) per animal species were collected using a stomach tube device before their morning feeding. The rumen fluid samples from each animal species were pooled, filtered through four layers of sheet cloth into a prewarmed thermos (39 °C), and immediately transported to the laboratory.

### Isolation, purification, and preparation of rumen-derived bacteria

The desired bacteria were enriched using a closed fermenter system (DURAN^®^ 250 mL glass screwcap bottle; Merck KGaA, Darmstadt, Germany) supplemented with KCN solution^35^. The incubation temperature was set at 39 °C with shaking at 120 rpm/min. The rumen fluid (10 mL) was first enriched for 4 days in nutrient broth (90 mL) and supplemented with 1 mL of a 10 g/L KCN solution. Then the enriched strains (10 mL) were inoculated into new nutrient broth (90 mL) supplemented with 1 mL of 20 g/L KCN solution and fermented for 4 days. This inoculation of nutrient broth with enriched bacteria was repeated three times, each time with a higher concentration of KCN solution (30–50 g/L). Next, the enriched strain (10 mL) was transferred to a mineral-glucose medium (90 mL) supplemented with 1 mL of 10 g/L KCN solution and fermented for 4 days. This step was repeated twice, each time with a higher concentration of KCN solution (20–30 g/L).

The nutrient broth was prepared and sterilized according to the manufacturer’s instructions (Difco Laboratories, Detroit, MI, USA). The mineral-glucose medium was first prepared by dissolving K_2_HPO_4_ (4.35 g), yeast extract (1 g), and trace element solution (10 mL of 0.3 g FeSO_4_·7H_2_O, 0.18 g MgSO_4_·7H_2_O, 0.13 g CoCl_2_, 0.04 g CaCl_2_, 0.04 g MnCl_2_·4H_2_O, and 0.02 g MoO_3_ per L) into 1 L distilled water. This medium was adjusted to pH 7.0 using hydrogen chloride (HCl) (0.1 N) before being autoclaved and supplemented with 2 mL glucose solution (500 g/L, an ampule for pharmaceutical intravenous infusion).

The final strain enrichment from the mineral-glucose medium (1 mL) was serially diluted with 9 mL NaCl solution (8.5 g/L) from 10^0^ to 10^−5^ to isolate 50–100 colonies/plate. An aliquot of each dilution (100 µL) was spread on three plates of nutrient agar and anaerobically incubated at 39 °C for 48 h (Memmert IF160; Memmert GmbH + Co. KG, Schwabach, Germany). The isolates were identified using stereomicroscopy at 40 × magnification and distinguished by colony appearance. The strain numbers were counted within animal species (buffalo had 8 strains, beef cattle had 21 strains, and dairy cows had 15 strains). The strains were purified twice by streaking on nutrient agar, and cryopreserved at − 80 °C.

For use in assays, the strains were defrosted at 4 °C and inoculated in nutrient broth (100 mL) supplemented with 1 mL KCN solution (10 g/L). Other than a 48 h incubation, the culturing and isolation conditions were the same. Each 30 mL of regrowth bacteria was centrifuged for 5 min at 4200*g* and 4 °C, washed twice, and resuspended in 10 mL of NaCl solution. The absorbance of the final pellet was adjusted to an optical density at 600 nm (OD_600_) of 1.0 using a spectrophotometer (UV/VIS Spectrometer; PG Instruments Ltd., London, UK). At OD_600_ = 1.0, the bacterial cell density in this study was 10^8^ colony forming units/mL.

### Rhodanese activity screening

The rhodanese activity present in each strain was screened in triplicate using a modified rhodanese assay as previously described^28,36^. The strain sample (10 mL at OD_600_ = 1.0) was pipetted into a 15 mL screwcap tube. After centrifugation for 10 min at 4200*g* and 4 °C, the supernatant was discarded and the cell pellet was resuspended in 10 mL TE-NaCl buffer, pH 7.6^28^. The sample underwent three sonication cycles on ice (60 s with 30 s cooling intervals, Transonic Digital S; Elma Schmidbauer GmbH, Singen, Germany). After centrifugation for 10 min at 4200*g* and 4 °C, the crude bacterial enzyme was pipetted (2.0 mL) into a 15 mL screwcap tube containing a reaction mixture (1.0 mL of 67 mM K_2_HPO_4_ and 1 mL of 100 mM Na_2_S_2_O_3_ in 200 g/L NaCl solvent). Then the sample was incubated at 39 °C for 10 min, supplemented with 1.0 mL KCN solution (100 mM in 200 g/L NaCl solvent), and incubated for another 30 min. Next, 500 µL formaldehyde solution (370 g/L) was added to the tube, followed by centrifugation for 10 min at 4200*g* and 4 °C. The supernatant (2 mL) was pipetted into a 15 mL screwcap tube containing 2 mL Fe(NO_3_)_3_ solution (150 g/L in 1.0 M HNO_3_) and incubated at room temperature for 10 min to form an intense red color at which point its absorbance was read at 460 nm. TE-NaCl buffer was used to calibrate the absorbance, both without and with the addition of potassium thiocyanate solution. Three blanks from each sample extract were included to omit the effects of elemental sulfur, for which the enzyme extracts were boiled for 40 min before undergoing the normal assay protocol. The protein content of the crude bacterial enzymes was measured by Lowry analysis^37,38^. The units of rhodanese activity are defined as µmol thiocyanate production/min/mg protein.

### Genetic identification

Total genomic DNA of the six strains was extracted, and the fragments were amplified by PCR (PCRmax Alpha Cycler 1; PCRmax Ltd., Staffordshire, UK). The following universal primers were used for amplification: 27F (5’-AGAGTTTGATCMTGGCTCAG-3’) and 1492R (5’-TACGGYTACCTTGTTACGACTT-3’). The thermal cycling conditions were denaturation at 95 °C for 5 min, followed by 32 cycles at 95 °C for 30 s, 55 °C for 30 s, and 72 °C for 1 min. A final extension was carried out at 72 °C for 7 min followed by cooling to 4 °C. The PCR products were purified using a spin column (ATGC Co. Ltd., Thailand). The 16S rRNA gene sample was sequenced with a genetic analyzer (ABI 3730xl 96-capillary DNA Analyzer; Applied Biosystems, Thermo Fisher Scientific, Waltham, MA, USA). The sequencing data were aligned with MEGA X^39^ using MUSCLE with default settings and then inspected for regions with high-quality alignment. The similarity of contigs (ranging from 1328 to 1430 bp) was analyzed using the BLAST search tool for species identification in GenBank.

Two phylogenetic trees were constructed using the 16S rRNA gene sequences. One tree was constructed for *E. faecium*, and the other for *E. gallinarum* with *Lactobacillus plantarum* WCFS1 (KC429782) as an outgroup. The nucleotide sequences of closely related strains were obtained from GenBank database. The analyses involved twelve closely related nucleotide sequences of *E. faecium* and eight closely related sequences of *E. gallinarum*. The trees were constructed using the Maximum Likelihood method and Tamura-Nei model^40^ with 10,000 bootstrap iterations in MEGA X. The initial trees for the heuristic search were automatically generated by applying the Neighbor-Join and BioNJ algorithms to a matrix of pairwise distances estimated using the Tamura-Nei model. The topology with the highest log likelihood value was then selected. The trees are drawn to scale, with branch lengths measured in the number of substitutions per site.

### Scanning electron microscopy

Cells were imaged at 150,000× magnification (FEI Helios Nanolab G3 CX; FEI Company, Hillsboro, OR, USA). A strain on MRS agar (2–3 colonies) was picked, added to 400 µL osmium tetroxide solution (10 g/L), incubated overnight at 4 °C, and centrifuged at 4200*g* for 3 min. The cell pellet obtained was resuspended in 1 mL of 30%, 50%, 70%, 80%, 90%, 95%, and 100% of ethanol/deionized water (v/v) for dehydration. Each dehydration reaction proceeded for 15 min at room temperature, after which the sample was centrifuged at 4200*g* for 3 min. The final pellet was resuspended in 100 µL ethanol, pipetted (10 µL) onto a filter membrane (Whatman^®^ Nuclepore™ Track-Etched Membranes, size 25 mm, pore size 0.2 μm), and left overnight in a desiccator. The filter membrane was glued to a stub and coated with gold (Cressington Sputter Coater 108auto; Cressington Scientific Instruments Ltd., Watford, UK).

### Biochemical test

Gram staining was performed using gentian violet as the primary stain and an iodine solution as the mordant, followed by ethanol as the decolorizer. Catalase activity was visualized as foam developed using a hydrogen peroxide and Triton X-100-based assay^41^. The starch hydrolysis test was performed by monitoring the purple iodine clear zone on nutrient agar plates containing amylose (10 g/L). Hydrogen sulfide gas production was tested using an iron nutrient broth. The strains were cultured overnight on MRS agar at 39 °C in or outside an anaerobic box (Sugiyamagen Ltd., Tokyo, Japan) to test growth under anaerobic and aerobic conditions, respectively.

Sugar fermentation after a 24 h incubation at 39 °C was conducted in triplicate using a modified MRS broth^42^ containing (per L): tryptone (10 g), yeast extract (5 g), beef extract (10 g), K_2_HPO_4_ (2 g), MnSO_4_·4H_2_O (0.05 g), MgSO_4_·7H_2_O (0.2 g), CH_3_COONa·3H_2_O (0.5 g), and C_6_H_14_N_2_O_7_ (2 g). After being autoclaved, MRS broth (2.25 mL) was added to different carbohydrate solutions (2.25 mL of 40 g/L ribose, glucose, lactose, fructose, and amylose), a strain sample (0.5 mL), and a pH indicator (5 mL of 20 g/L sterile bromocresol purple solution).

The final pH, cell density, organic acids, and ammonia nitrogen were analyzed after 24 h of fermentation in MRS broth (Difco Laboratories). The pH was measured using a pH meter, and the cell density was evaluated at 600 nm (UV/VIS Spectrometer). The concentrations of lactic acid and volatile fatty acids were determined using a gas chromatograph (Nexis GC-2030; Shimadzu Co., Kyoto, Japan). Each gas chromatography sample was pre-treated with a periodic acid reagent in a 1.5 mL vial^43^. The ammonia nitrogen concentration was measured at 630 nm (UV/VIS Spectrometer)^44^.

The growth at different pH, temperatures, and CN substrate concentrations was assayed in triplicate using MRS broth (Difco Laboratories). For pH variations (4–10), MRS broth (90 mL) was adjusted to pH levels using HCl and NaOH solutions, and supplemented with a strain (10 mL). At varied temperatures, MRS broth prepared without adjustments was used as the medium. For KCN concentrations between 0 and 400 mg/L of fermentation, MRS broth was supplemented with different KCN solutions (1 mL) before being strained. The culturing conditions were incubated for 24 h with agitation at 120 rpm/min.

### CN-thiocyanate transformation assay

We developed a CN-thiocyanate transformation assay by using a modified in vitro rumen fermentation technique^45^. This assay was conducted in closed vessels (DURAN^®^ 250 mL glass bottle) containing KCN as the source of CN compounds. Phosphate-buffered saline (75 mL of 16.0 g NaCl, 0.4 g KCl, 2.84 g Na_2_HPO_4_, and 0.48 g KH_2_PO_4_ per L), cell-free rumen fluid (45 mL), distilled water (10 mL), and micromineral solution (12 µL)^45^ were added to each sterile vessel. The rumen fluid was obtained from two fistulated dairy cows. The pH of all cell-free rumen fluid mediums was adjusted to 7.4 before being autoclaved. The medium was supplemented with 2 mL glucose solution (500 g/L), 2 mL Na_2_S_2_O_3_ solution (300 g/L), 1 mL KCN solution (28.92 g/L), and 15 mL distilled water without (baseline) or with strains. The strain numbers were 10^7^ colony forming units/mL of incubation, and the thiocyanate formation availability was 80 mg HCN equivalent/L. The vessels were purged with CO_2_ gas, the caps were screwed on, and they were incubated at 39 °C with shaking at 120 rpm/min. Three vessels from baseline and three vessels from each strain were withdrawn at 3, 6, 12, 24, and 48 h of incubation. The total CN concentration was immediately analyzed using the distillation method described by Fisher and Brown^46^. Various concentrations of KCN solution were used as standards. CN compounds were measured in mg HCN equivalent/L. The cell density was evaluated at 600 nm. The volatile fatty acid production was analyzed using gas chromatography (Nexis GC3-2030; Shimadzu). Spectrophotometry was conducted to examine the concentrations of ammonia nitrogen^44^ and glucose^47^.

### Statistical analysis

The data for growth and CN-thiocyanate transformation were analyzed using analysis of variance (ANOVA) in SAS (v6.12)^48^ with the following model:

$${\text{Y}}_{{{\text{ij}}}} =\upmu + \upalpha _{{\text{i}}} +\upvarepsilon _{{{\text{ij}}}} ,$$

where Y_ij_ = observation, μ = overall mean, α_i_ = strain effect (i = 6 strains, or i = 6 strains + 1 baseline), and ε_ij_ = error. The differences among strain means were assessed with the Duncan’s test with *p* ≤ 0.05. Pearson’s correlation of thiocyanate degradation and strain fermentation function for 12–48 h of incubation was analyzed using the CORR procedure. The correlation heatmap was analyzed using the CLUSTER procedure.

## Data Availability

The data of 16S rRNA gene sequences from this study are uploaded to NCBI, and are available to access from https://www.ncbi.nlm.nih.gov/nuccore/?term=MZ959826:MZ959831 [accn].
